# High-fidelity light-field display with enhanced information utilization by modulating chrominance and luminance separately

**DOI:** 10.1038/s41377-025-01752-x

**Published:** 2025-02-10

**Authors:** Zhaohe Zhang, Xunbo Yu, Xin Gao, Boyang Liu, Hanbo Wang, Chao Gao, Zeyu Hao, Ruiang Zhao, Xinzhu Sang

**Affiliations:** 1 State Key Laboratory of Information Photonics and Optical Communications, Beijing University of Posts and Telecommunications (BUPT), 100876 Beijing, China; 2 Jianghuai Advance Technology Center, Hefei, 230000 China; 3 Zhuhai Zhenxiang Optoelectronics Technology Co., Ltd, Zhuhai, 519075 China

**Keywords:** Displays, Imaging and sensing

## Abstract

Light-field displays typically consist of a two-dimensional (2D) display panel and a light modulation device. The 2D panel presents synthesized parallax images, with the total information content of the three-dimensional (3D) light field dictated by the panel’s total resolution. Angular resolution serves as a critical metric for light-field displays, where higher angular resolution correlates with a more realistic 3D visual experience. However, the improvement of angular resolution is typically accompanied by a reduction in spatial resolution, due to the limitations of the 2D display panel’s total resolution. To address this challenge, a light-field display method with enhanced information utilization is introduced, achieved through the independent modulation of chrominance and luminance. A static light-field image display system is proposed to verify the feasibility of this method. The system employs a bidirectional angular modulation grating (BAMG) and a collimated light source (CLS) to create uniformly distributed viewpoints in space. A luminance modulation film (LMF) and a chrominance modulation film (CMF) are utilized to modulate the light-field information, with chrominance and luminance synthesized images printed at pixel densities of 720 pixels per inch (PPI) and 8000 dots per inch (DPI), respectively, to align with the differential sensitivities of the human visual system. In the experiment, the proposed display system achieves a full-parallax, high-fidelity color display with a 98.2° horizontal and 97.7° vertical field of view (FOV). So, the light-field display method of modulating chrominance and luminance separately has been proven to achieve high-fidelity display effects.

## Introduction

3D display technologies, including volumetric displays, integral imaging and holographic displays offer various approaches to achieving more realistic visual effects than traditional 2D displays^1–4^. Among these, light-field displays, in particular, hold significant promise for creating high-fidelity visual experiences. Light-field displays typically consist of a 2D display panel coupled with a light modulation device. The 2D panel presents synthesized parallax images, with the total information content of the 3D light field determined by the display panel’s space-bandwidth product (SBP), often reflected in its total resolution. The light modulation device controls the formation of spatial viewpoints, enabling the 3D effect. Techniques such as lenticular lens array, diffraction gratings, and liquid crystal lenses are used to manipulate the light field^5–7^. However, they do not alter the total information content, which remains constrained by the display panel’s total resolution.

The key performance metrics of light-field displays include angular resolution, spatial resolution, and viewing angle. Angular resolution is typically expressed in parallax images per degree (PIPD), which indicates the information density^8–10^. In conventional light-field encoding methods, each pixel on the 2D panel corresponds directly to a pixel in the parallax image for each viewing angle. As a result, these three metrics—angular resolution, spatial resolution, and viewing angle—are all constrained by the total resolution of the 2D panel. The total resolution can be understood as the product of the spatial resolution, the horizontal information density, the horizontal viewing angle, the vertical information density, and the vertical viewing angle. The viewing angle is determined by the light modulation device, while the spatial resolution need at least to meet the threshold required for human visual acuity. Higher information density approximates the display effect to a true light field. However, due to the limited total resolution of 2D panels, the information density in these displays falls short, resulting in a less realistic visual experience.

To increase the information density in light-field displays, efforts have focused on increasing the SBP. Guozhen Liang et al. improved SBP through micrometer-scale phase modulators, refining optical components^11^. Rui Chen et al. and Zhuoran Fang et al. further advanced pixel density with hybrid photonic systems and graphene-assisted platforms, respectively^12,13^. Additionally, time-division multiplexed directional backlighting also enhances SBP by leveraging temporal redundancy. Masaki Yamauchi et al. and Boyang Liu et al. developed systems that combine high-speed projection with multiplexing to expand SBP^14,15^. Moreover, multi-screen tiling, which expands the display area, has been explored to boost SBP. Lixia Ni et al. and Wang Qionghua et al. utilized multi-projection systems and spliced LCD panels to enhance the overall amount of information^16,17^. Finally, layering multiple display panels has shown promise in enhancing SBP by stacking resolution layers. Gordon Wetzstein et al. introduced tensor displays with multilayer setups, while Liming Zhu et al. employed multi-plane projection to maintain brightness and image quality across stacked layers^18,19^. In summary, while increasing the SBP of display devices can improve the information density and overall quality of light-field displays, it often results in more complex and cumbersome systems, thus making light modulation and system integration more challenging.

Another approach to optimizing the efficiency of 3D light field displays involves adapting information density based on the visual characteristics of the human eye. Leveraging the concept of foveated vision, Hua et al. proposed a glasses-free 3D display that employs a large-scale 2D metagrating complex to deliver high-resolution content in the foveal region while reducing resolution in peripheral areas, thus maximizing the effective use of the display’s resources^8^. Similarly, Cem et al. developed a foveated near-eye display using computational holography that dynamically adjusts the FOV to follow the user’s gaze, enhancing the efficiency of SBP without requiring mechanical components^20^.

Despite these advances, achieving high-fidelity light-field displays remains a significant challenge with current technological solutions. One of the primary obstacles is maintaining high spatial resolution and information density across wide viewing angles. For instance, a full-parallax display with horizontal and vertical viewing angles approaching 100°, a spatial resolution of 1000 × 1000 pixels, and an information density of 1 PIPD at a viewing distance of 1.1 meters would necessitate a display panel resolution of approximately 100,000 × 100,000 pixels. Implementing this over a 12.5-inch × 12.5-inch display area would require a pixel density of about 8000 PPI. PPI is suitable for grayscale images, where each monochrome pixel has 256 shades of gray. DPI refers to the number of dots per inch, while PPI refers to the number of pixels per inch. DPI is suitable for black-and-white binary images, where a dot represents a pixel with a grayscale of 2. The difference in information quantity between the two is a factor of 8.

Traditional display technologies are inadequate to meet these demands due to the limitations imposed by current manufacturing precision, which makes it challenging to uniformly maintain such high pixel densities across the entire display.

Here, a light-field display method with enhanced information utilization is proposed by independently modulating chrominance and luminance, and a static light-field image display system is constructed to verify the feasibility of this method. At the same time, the design and analysis of the dynamic light-field display device constructed using this method were also conducted.

The perception of chromatic and luminance information in the human eye operates independently, with a much higher resolution for luminance compared to chromatic information^21–24^. This inherent difference in visual perception has been practically applied in various technologies, particularly in the field of video compression and television broadcasting. For instance, in the YUV color space used in video compression, luminance (Y) and chrominance (U and V) signals are processed separately. Because the human eye is more sensitive to brightness details than to color details, chrominance signals can be compressed more heavily than luminance without significantly affecting perceived image quality. This principle is widely utilized in compression standards such as JPEG, MPEG, and H.264/AVC, where chrominance is often stored or transmitted at a lower resolution than luminance, thereby reducing data size while maintaining visual fidelity. A design method for a high-fidelity light-field display system is proposed, where chrominance information is allocated a lower pixel density to optimize information utilization efficiency, while luminance information is assigned a higher pixel density to ensure exceptional high-fidelity display performance.

**Fig. 1:**
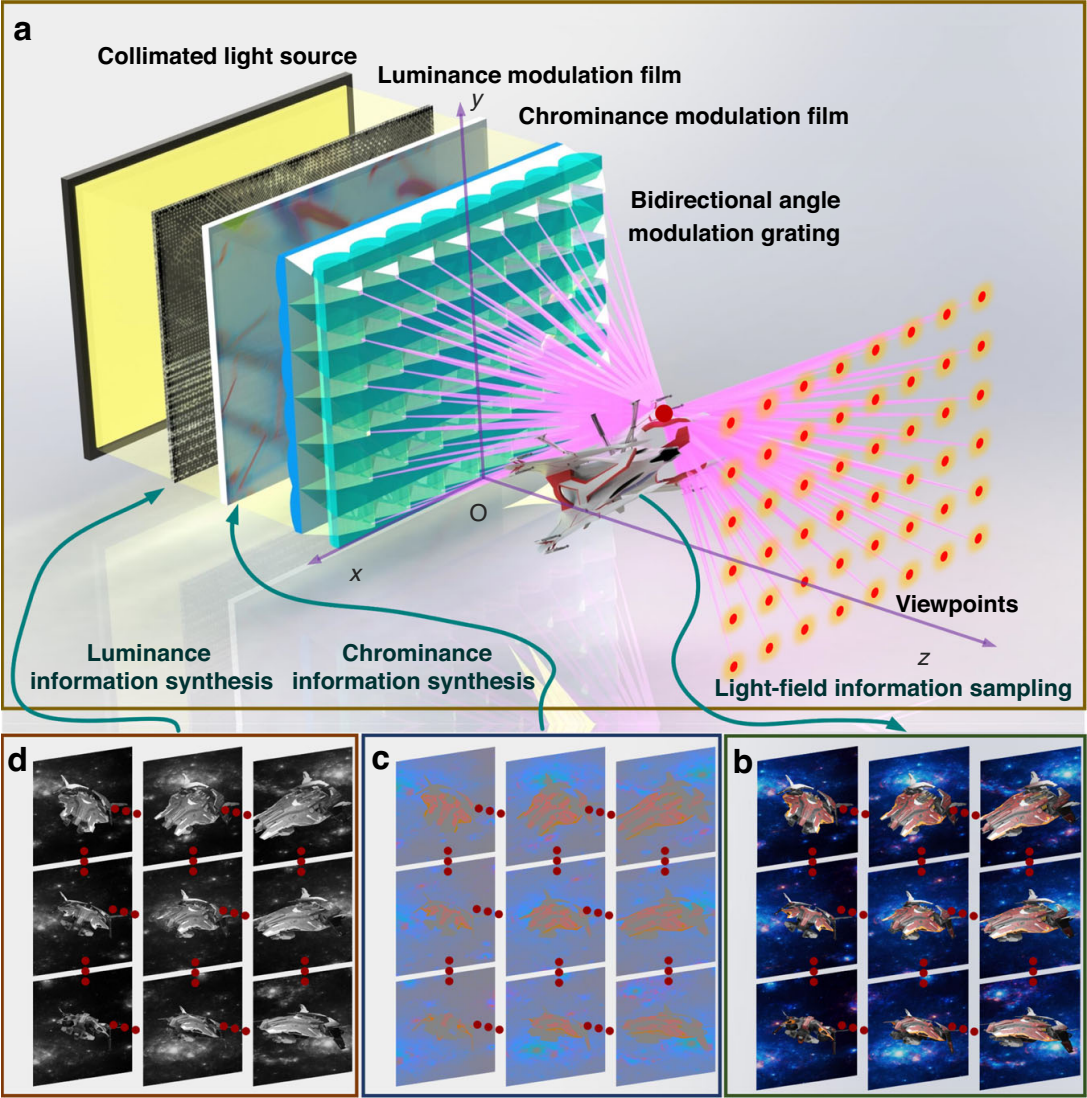
Schematic of the high-fidelity static light-field image display system with separated chrominance and luminance information. **a** System structure and optical path diagram. The “spaceship” model represents the intended 3D light-field image, with the pink lines illustrating the light paths used by the light modulation units to construct viewpoints. The red dots indicate the generated viewpoints. **b** Parallax image array schematic. Each image represents the parallax view observed from different viewpoints, used for information processing. The red dots signify other parallax images that are omitted between two displayed images. **c** Chrominance parallax image array. Each image represents the chrominance information extracted from the parallax image array, used to synthesize the chrominance image displayed on the CMF in **a**. **d** Luminance parallax image array. Each image represents the luminance information extracted from the parallax image array, used to synthesize the luminance image displayed on the LMF in **a**

The static light-field image display system utilizes a BAMG and a CLS to establish uniformly distributed viewpoints. In the system, the CMF, designed to modulate chrominance information, can be produced through inkjet printing methods, while the LMF, intended to modulate luminance information and requiring high pixel density, can be fabricated using laser exposure techniques. The chrominance synthesis image projected onto the CMF can be derived from the chrominance parallax image array (Fig. 1c) using beam backtracking. Similarly, the luminance synthesis grayscale image can be obtained from the luminance parallax image array (Fig. 1d) through the same beam backtracking process. However, the laser exposure process produces binary black-and-white exposure points, without the capability to adjust grayscale.

As a result, the LMF can only display the luminance synthesis as a halftone image. The human eye exhibits spatial summation properties, meaning that it does not perceive brightness changes on a pixel-by-pixel basis, but rather integrates information from different pixels over a certain spatial area to form a perception of the overall image^25,26^. In the field of image processing, this characteristic is widely used in halftone processing, where a dither matrix converts continuous-tone grayscale images into binary or limited grayscale images, while still allowing the human eye to perceive the original grayscale variations. Due to the correlation between the information of adjacent luminance parallax images and the information of neighboring pixels within the same luminance parallax image, a discrete dither matrix is proposed. By leveraging the spatial summation properties of the human eye, it can convert a luminance synthesis grayscale image into a halftone image. This method enhances the information utilization efficiency of light-field display technology. As a result, the experiment demonstrated a full-parallax, high-fidelity color light-field display with a horizontal FOV of 98.2° and a vertical FOV of 97.7°, achieving a spatial resolution of 1000 × 1000 and a display depth of 45 cm on a 12.5-inch × 12.5-inch screen.

Apart from being used for static displays, the light-field display method proposed in this paper also has potential in the field of dynamic displays. Producing high-pixel-density color grayscale dynamic display screens is extremely challenging, which is one of the key obstacles to the development of light-field displays. However, the proposed display method demonstrates an alternative approach to dynamic light-field display: separating chrominance control and luminance control onto two screens. A low grayscale luminance modulation screen is used to reduce computational load and circuit complexity, allowing for higher pixel density. A low pixel density chrominance modulation screen is employed to reduce system integration difficulty and cost. By integrating the two screens, high-quality dynamic light-field display effects can be achieved.

## Results

### Static light-field image display system with separate encoding of chrominance and luminance

Figure 1 illustrates the system architecture of the proposed full-parallax high-fidelity light-field display, which consists of a CLS, an LMF, a CMF, and a BAMG. The LMF modulates the light field’s luminance, while the CMF modulates its chrominance. The BAMG directs the collimated light beams to form uniformly distributed viewpoints in space. The use of a CLS, rather than a conventional divergent source, is crucial to prevent defocusing, as luminance and chrominance are independently controlled by distinct films.

During the implementation, the parallax images array of the displayed content is separated into luminance parallax images array and chrominance parallax images array through chrominance and luminance separation. Given that the perception of chromatic and luminance information in the human eye operates independently, with a much higher resolution for luminance than for chromatic information, the luminance and chrominance disparity image arrays are synthesized at different pixel densities. The LMF and CMF are then produced by printing the synthesized luminance and chrominance information, as depicted in Fig. 1.

### Design of the full parallax optical system

**Fig. 2:**
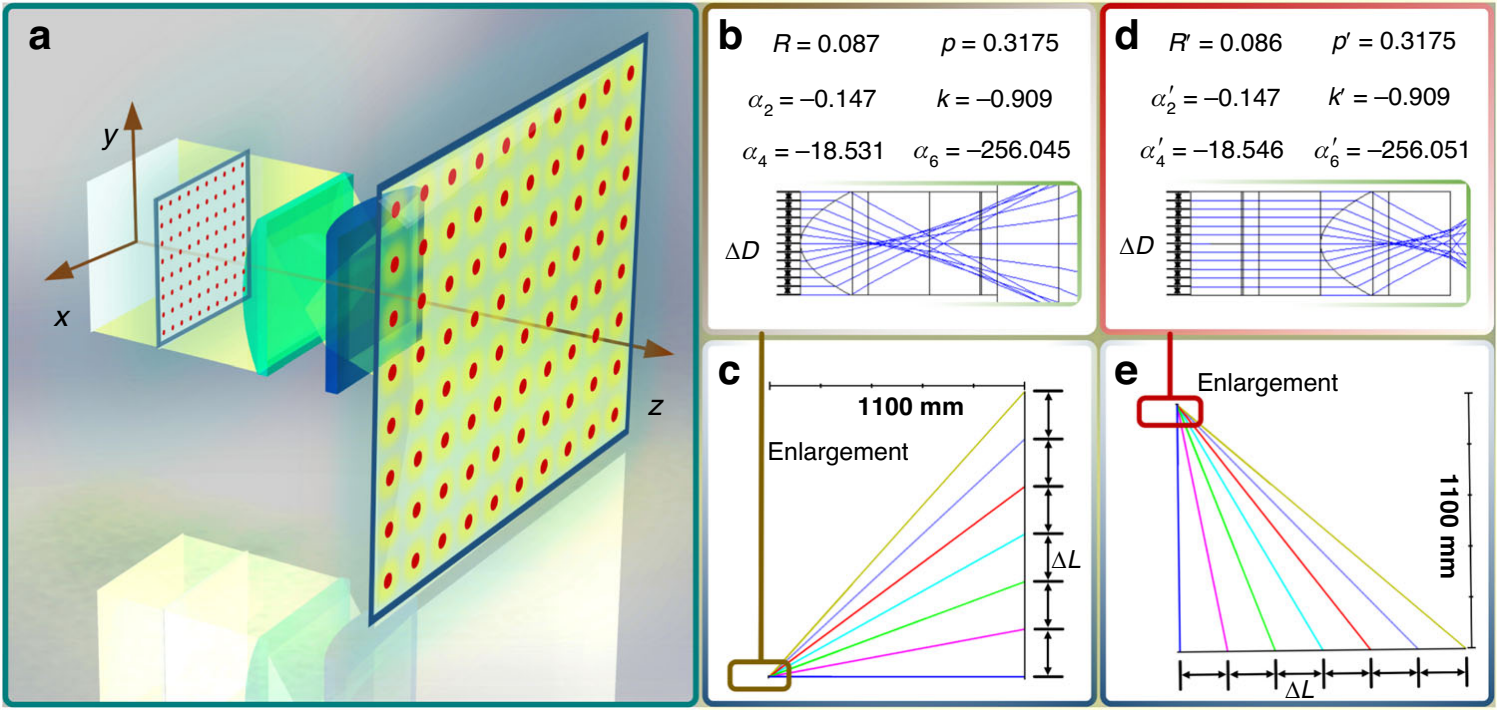
Schematic of the optical design principles for the light control unit used to construct uniformly distributed viewpoints in space. **a** Structural diagram and optical path of the light control unit. The small boxed area on the left represents the object plane, where small red dots indicate equidistant object points on the plane. The larger boxed area on the right represents the image plane, where object points from the object plane are mapped through the optical structure to form image points. The large red dots denote these image points on the image plane. **b** Optical design and parameters of the light control unit in the y-z plane. The thin blue lines represent the collimated incident light beams entering the object plane at equal intervals. **c** Schematic of the FOV constructed by the light control unit in the positive y-axis of the y-z plane. The colored lines represent the outgoing light paths corresponding to the equidistant incident beams. **d** Optical design and parameters of the light control unit in the x-z plane. The thin blue lines represent the collimated incident light beams entering the object plane at equal intervals. **e** Schematic of the FOV constructed by the light control unit in the positive x-axis of the x-z plane. The colored lines represent the outgoing light paths corresponding to the equidistant incident beams

The high-fidelity static light-field image display system is engineered to provide a 100-degree viewing angle in both horizontal and vertical directions at a viewing distance of 1.1 meters. Considering that human visual acuity is approximately 1 arcminute, the pitch of the horizontal and vertical lenticular lenses is designed to be 0.3175 millimeters, as shown in Fig. 2. The pixel density of the intensity-modulated film reaches 8000 DPI, with a selected BAMG of 80 LPI. Calculations show that the system has 100 parallax images. According to the analysis of information density in the literature, when the information density reaches 1 PIPD^27,28^, a realistic display effect can be achieved. Therefore, the viewing angle of this display system is designed to be 100°. In the preparation of light control devices and during the system assembly process, errors may occur. To ensure that the information density exceeds 1 PIPD, an intentional tolerance biasing strategy is applied to the viewing angle during manufacturing. Thus, the actual measured parameters of the horizontal viewing angle and vertical viewing angle are 98.2° and 97.7°. To ensure that the collimated light passes through horizontal and vertical cylindrical lenses to form uniformly distributed viewpoints, aspheric optical structures are employed. The optimization results and parameters of the aspheric optical structures are presented in Fig. 2. The vertical FOV of the unit is depicted in the y-z plane FOV map, with the magnified light path details shown in the y-z plane light path diagram. Similarly, the horizontal FOV is illustrated in the x-z plane FOV map, with the corresponding magnified light path details in the x-z plane light path diagram. The optimization results in both horizontal and vertical directions can be summarized as follows: “The light beams incident at the same distance ∆*D* pass through the lens and reach the position of the image plane at the same distance ∆*L*.” Here, *R* denotes the design value of the curvature radius for the vertical light control lens, *p* represents the pitch, *k* indicates the conic constant, and *α*_2_, *α*_4_, and *α*_6_ refer to the second-, fourth-, and sixth-order aspherical coefficients, respectively. Similarly, $p^{\prime}$, $R^{\prime}$, $k^{\prime}$, ${\alpha }_{2}^{\prime}$, ${\alpha }_{4}^{\prime}$ and ${\alpha }_{6}^{\prime}$ correspond to the parameters for the horizontal light control lens. The lenses designed with these parameters, as outlined in Fig. 2, enable the construction of a wide-angle full-parallax light field with uniformly distributed viewpoints, providing a solid physical foundation for the light-field encoding algorithm. In addition, the simulation results show that the root-mean-square radius of the diffused spot is smaller than the pixel size in the full FOV, which means that the chromatic aberration in the display effect is almost invisible to the human eye.

### The coding method for chrominance and luminance

**Fig. 3:**
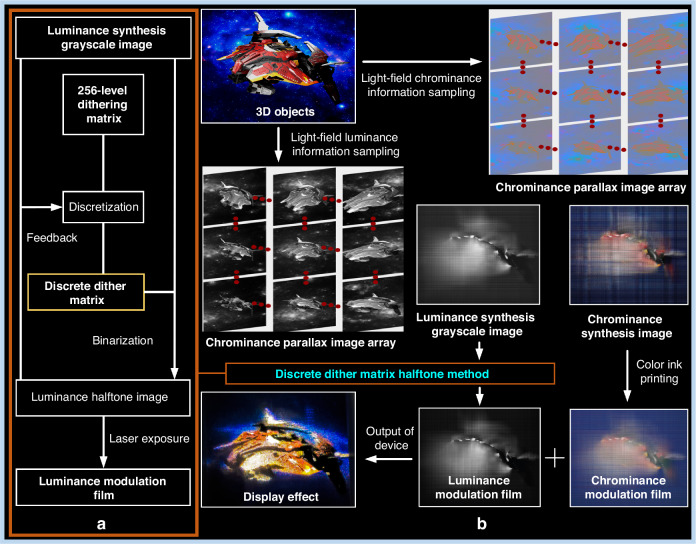
Light-field display encoding algorithm flowchart. **a** Discrete dither matrix halftone method flowchart. Discretize the 256-level dithering matrix to generate a discrete dither matrix. Use the discrete dither matrix to binarize the luminance synthesis grayscale image, creating a luminance halftone image. The degree of discretization of the dither matrix depends on the feedback from the fitting of the luminance halftone image to the luminance-composite grayscale image. The luminance halftone image is prepared using laser exposure technology to form a luminance modulation film. **b** Overall flowchart of the light-field display. Three-dimensional objects undergo light-field information sampling to generate arrays of chromatic disparity images and luminance disparity images, respectively. The luminance disparity image array produces a luminance-composite grayscale image, while the chromatic disparity image array generates a chrominance synthesis image. The luminance-composite grayscale image is prepared using the separated dither matrix halftoning method to create a luminance modulation film. The chrominance synthesis image is produced through color ink printing to create a chrominance modulation film. These two films provide light information for light-field display

**Fig. 4:**
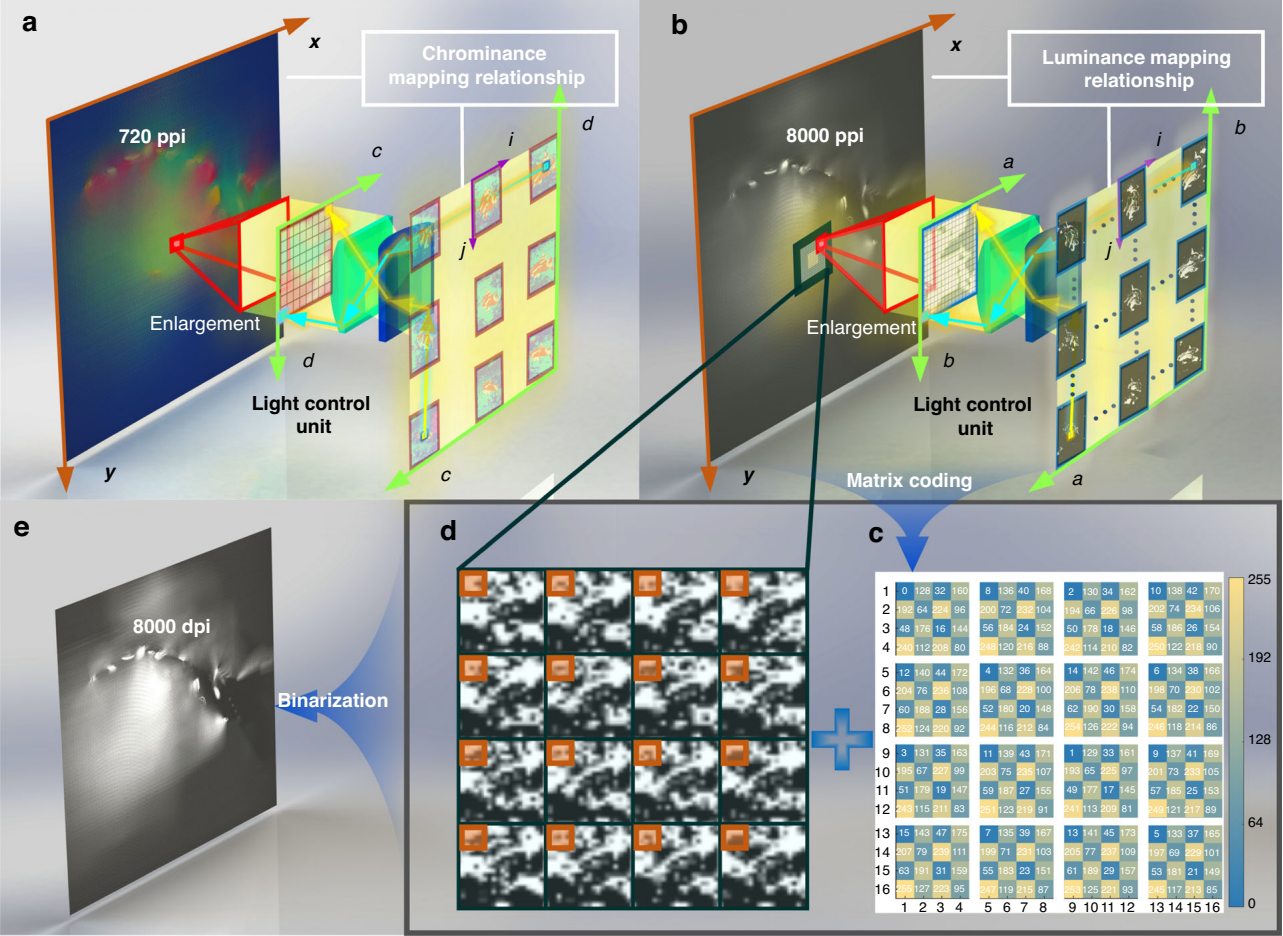
Schematic of the light-field display information synthesis process. **a** Schematic of chrominance information synthesis. The large color image on the left, with the x-y coordinate system, represents the 720 PPI chrominance synthesis image. The grid image in the center shows an enlarged section of the chrominance synthesis image. The array of images on the right represents the chrominance parallax image array. The arrows near the lens illustrate the pixel mapping relationship between the chrominance parallax image array and the chrominance synthesis image. **b** Schematic of luminance grayscale information synthesis. The large grayscale image on the left, with the x-y coordinate system, represents the 8000 PPI luminance synthesis grayscale image. The grid image in the center shows an enlarged section of the luminance synthesis grayscale image. The array of images on the right represents the luminance parallax image array. The arrows near the lens illustrate the pixel mapping relationship between the luminance parallax image array and the luminance synthesis grayscale image. **c** Discrete dither matrix. The discrete dither matrix contains 256 elements, organized into 4 × 4 submatrices. The area outlined by orange lines represents the 4 × 4 submatrices, with each submatrix containing 16 elements. The elements in the matrix are integers ranging from 0 to 255, representing the threshold values used in the binarization process, corresponding to a color gradient from blue to yellow. **d** Enlarged view of the luminance synthesis grayscale image within a 4 × 4 light control unit. The large area outlined by dark lines represents a synthesized encoded image within a single light control unit. The smaller area outlined by orange lines contains 4 × 4 pixels, corresponding to the submatrix highlighted in **c**. The binarization process using the discrete dither matrix for the luminance synthesis grayscale image can be described as follows: Each pixel within the orange-outlined area is compared against the corresponding threshold value in the submatrix; if the pixel’s grayscale value exceeds the threshold, it is binarized to 1; otherwise, it is set to 0. The orange-outlined area is then shifted to the next region for subsequent binarization. **e** 8000 DPI luminance synthesis halftone image. This image represents the halftone image obtained after binarizing the luminance synthesis grayscale image using the discrete dither matrix, which can be used for LMF fabrication

Figure 3 presents an overall flowchart of the coding method for chrominance and luminance. Specifically, Fig. 3a illustrates the process of the discrete dither matrix halftone method, while Fig. 3b shows the overall flowchart of the light-field display system. Figure 4a and Fig. 4b illustrate the process of synthesizing luminance and chrominance encoding images using the luminance parallax image array and the chrominance parallax image array. According to the reverse-tracing algorithm, the mapping relationship between the encoded images and the parallax image arrays is as follows:

1 $$\begin{array}{c}V{p}_{(c,d)}^{(a,b)}(i,j)=\left(\begin{array}{c}LI((mi+a){d}_{L},(nj+b){d}_{L}),\\ CI((ki+c){d}_{C},(lj+d){d}_{C})\end{array}\right)\end{array}$$

Here, ${{V}_{p}}_{\left(c,d\right)}^{\left(a,b\right)}(i,j)$ represents the viewpoint information located within the $i-{\rm{th}}$ cylindrical lens in the horizontal direction and the *j*-*th* cylindrical lens in the vertical direction, corresponding to the (*a,b*) luminance parallax image and the (*c,d*) chrominance parallax image. *LI*(*x,y*) represents the distribution of luminance information, while *CI*(*x,y*) represents the distribution of chrominance information. The variable *m* denotes the number of columns in the luminance parallax image array, *n* denotes the number of rows, *k* represents the number of columns in the chrominance parallax image array, and *l* denotes the number of rows. *d*_*C*_ and *d*_*L*_ refer to the pixel sizes of the chrominance and luminance synthesis images, respectively.

Considering the grating density of 80 LPI and the limitations of the manufacturing process, a LMF with a pixel density of 8000 DPI was selected. Effect testing of CMFs with different pixel densities showed that when the luminance modulation film is set to 8000 DPI, the appropriate pixel density for the chromatic modulation film is 720 PPI. By applying a beam reverse-tracing algorithm, the 100 × 100 array of luminance parallax images can be encoded into an 8000 PPI luminance synthesis grayscale image, which is then converted into an 8000 DPI luminance synthesis halftone image using a discrete dither matrix halftoning algorithm. Consequently, the parameters in the equation must satisfy the following quantitative relationship:

2 $$a\in [0,100),b\in [0,100),c\in [0,9),d\in [0,9),i\in [0,1000)$$

3 $$j\in [0,1000),m=n=k=l=1000,\frac{d}{b}=\frac{c}{a}=\frac{{d}_{L}}{{d}_{C}}$$

However, common pixel fabrication techniques such as liquid crystal pixel technology, inkjet printing, and micro-LED fabrication are inadequate for producing display panels with a pixel density of 8000 PPI and a total resolution of 100,000 × 100,000 pixels. Although laser exposure technology cannot directly create grayscale-adjustable exposure points, it can generate black-and-white exposure points with a diameter of less than 3.175 µm, enabling the production of an 8000 DPI binary halftone luminance synthesis image. Therefore, the 8000 PPI grayscale luminance synthesis image, composed of 256 grayscale levels, must be converted into a binary halftone image before it can be fabricated using laser exposure technique.

### Performance

**Fig. 5:**
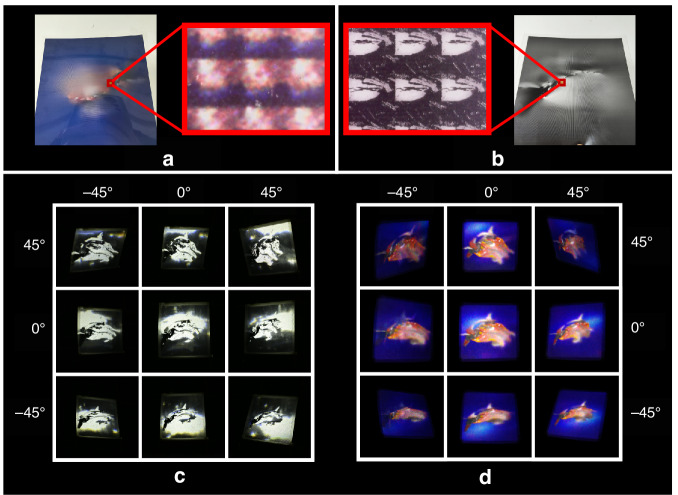
Physical images of the CMF and LMF and Captured images of the light-field display effects. **a** Physical image of the CMF: The left image shows a macroscopic view of the CMF, while the right image provides a close-up of its microscopic details. **b** Physical image of the LMF and its magnified details: The left image presents a macroscopic view of the LMF, and the right image offers a close-up of its microscopic structure. **c** Light-field display effect using the LMF as an independent 2D display panel (see visualization 1). The nine images were captured from different viewing angles. **d** Light-field display effect using the CMF as an independent 2D display panel (see visualization 2). The nine images were captured from different viewing angles

A color printer is used to print the chrominance synthesis image at 720 PPI, resulting in a chrominance modulation film. A laser typesetting machine is employed to print the luminance synthesis image at 8000 DPI, producing a luminance modulation film. As shown in Fig. 5, because the chrominance synthesis image is printed with low-density pixels, the elemental images on the chrominance modulation film appear blurry. In contrast, since the luminance synthesis image is printed with high-density pixels, the elemental images on the luminance modulation film are clear.

This method of using different pixel densities for luminance and chrominance information is well-suited to the perceptual characteristics of the human eye regarding these types of information. In the display system depicted in Fig. 1, with only the chrominance modulation film retained, the display effect is as shown in the figure and video. In this mode, the color contours of 3D images are displayed. With only the luminance modulation film retained, the display effect is also shown in the figure and video. In this mode, the brightness details of the 3D image are clearly visible. When both the luminance modulation film and chrominance modulation film are used simultaneously, the brightness details and color information of the 3D image are well restored. In the experiment, the proposed static light-field image display system achieves a full-parallax, high-fidelity color light-field display with a 98.2° horizontal and 97.7° vertical FOV.

## Discussion

Here, a high-fidelity light-field display method is introduced, featuring enhanced information utilization through the independent modulation of chrominance and luminance. During the production of the luminance and chrominance films, the human eye’s differential sensitivity to chrominance and luminance information is taken into account. A 9 × 9 chrominance parallax image array is used to generate a chrominance synthesis image, which is then printed at 720 PPI using a multi-color inkjet printer to produce the chrominance film. Conversely, recognizing the human eye’s high sensitivity to luminance information, a 100 × 100 luminance parallax image array is employed to generate a luminance synthesis image at 8000 PPI. Due to the correlation between adjacent luminance parallax images and neighboring pixels, a discrete dither matrix is proposed to convert the 8000 PPI grayscale image into an 8000 DPI halftone image. This luminance synthesis image is then printed using a laser typesetting machine with 8000 DPI to produce the luminance film. Experimental results demonstrate that when only the LMF is retained, the brightness details of the 3D image are clearly visible, whereas with only the CMF, the color contours of the 3D images are effectively displayed. This outcome aligns with the characteristics of human visual perception regarding chrominance and luminance. When both the LMFs and CMFs are employed simultaneously, the brightness details and color information of the 3D image are well restored. If the display reaches a pixel density of 8000 PPI, its information quantity per square inch (IQPSI) will reach 183.11 MB. If it achieves the display area of 12.5 inches × 12.5 inches proposed in the text, its total information quantity will reach 27.98 GB.

**Fig. 6:**
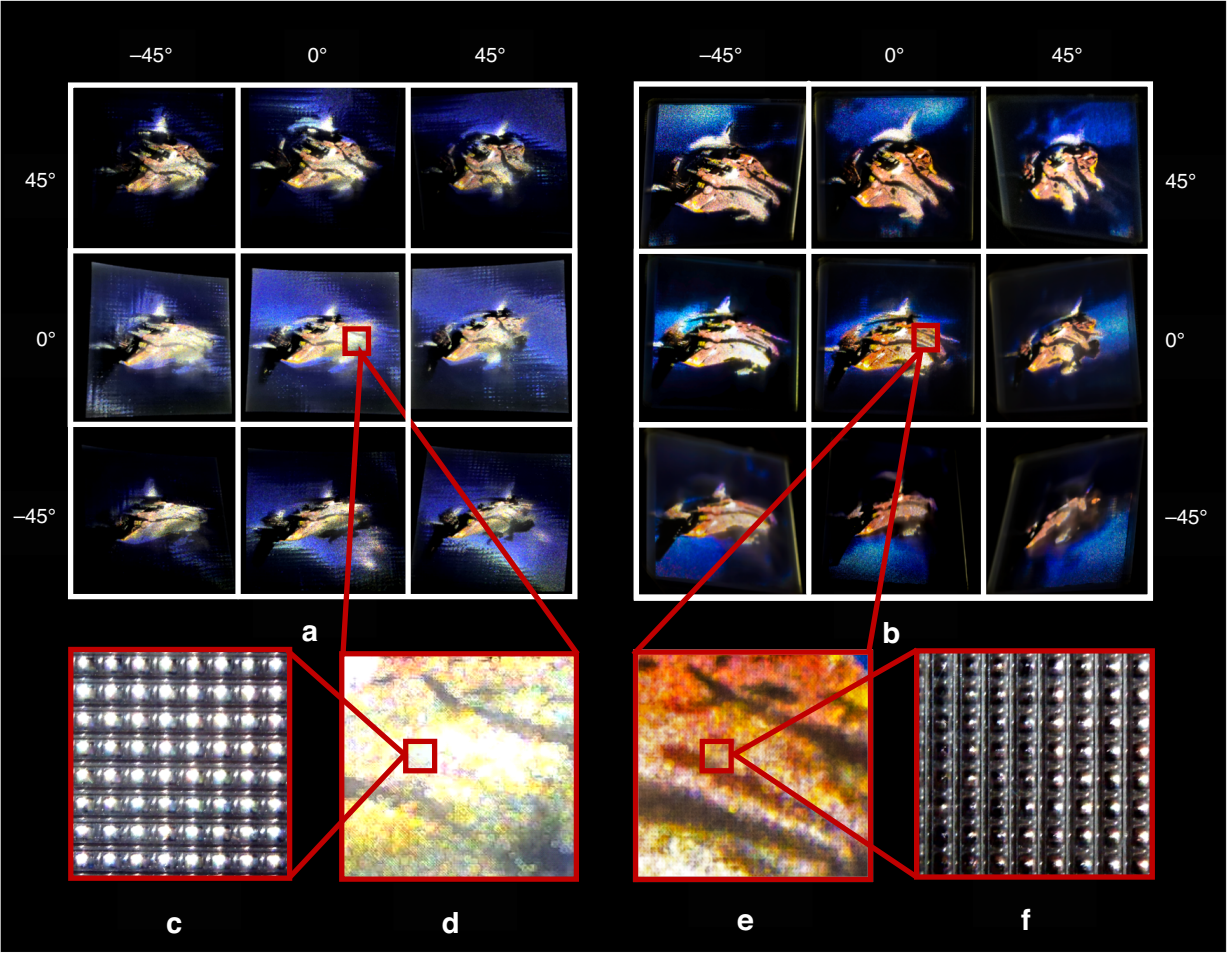
The display effect diagram of the proposed static light-field image display system and the comparison diagram of its display effect with traditional methods. **a** The display effect diagram of the traditional light-field display method with a pixel density of 1784 PPI, under the premise of controlling variables. The nine images were captured from different viewing angles. **b** Microscopic image of the display unit using traditional methods. **c** Enlarged display effect diagram of the traditional method. **d** Display effect diagram of the proposed static light-field image display system (see visualization 3). **e** Enlarged display effect diagram of the static light-field image display system. **f** Microscopic image of the display unit in the static light-field image display system

The static light-field image display system constructed in this paper uses an 8000 DPI (IQPSI 7.63MB) LMF and a 720 PPI (IQPSI 1.48MB) CMF, with an IQPSI of 9.11 MB, equivalent to a pixel density of 1784 PPI (IQPSI 9.11MB) for a traditional light-field display. Figure 6 illustrates the difference in display effects between the traditional light field display method and the proposed light field display method by modulating chrominance and luminance separately, under controlled variables and with the same information quantity. Figure 6c and Fig. 6e present magnified views of the display effects of the two display methods at the same viewpoint and position. Based on the comparison of display effects, it can be concluded that the light field display method by modulating chrominance and luminance separately improves information utilization efficiency.

**Fig. 7:**
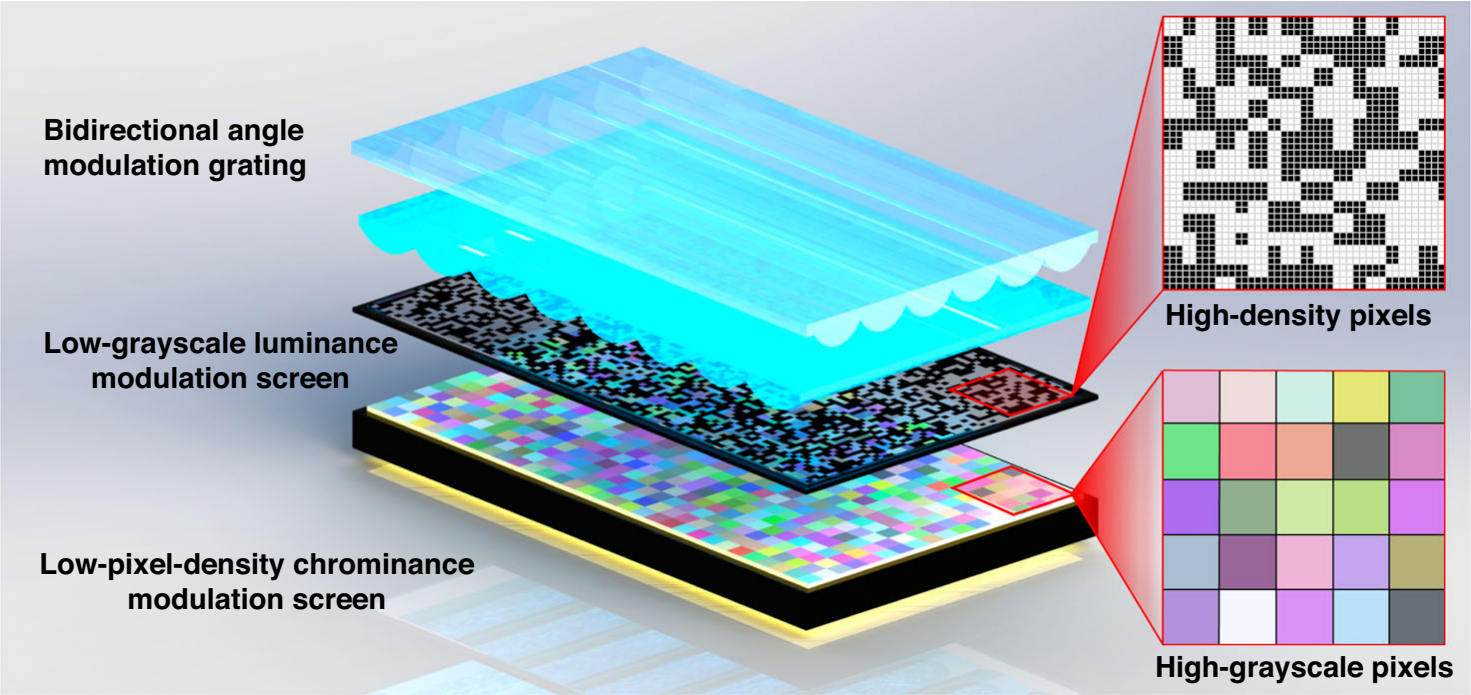
Schematic diagram of dynamic light-field display with separated chrominance and luminance modulation. The low-gray-level luminance modulation screen and the low-pixel-density chrominance modulation screen separately control the luminance and chrominance information for dynamic light field displays. The advantage of the low-gray-level luminance modulation screen is its high pixel density, while the advantage of the low-pixel-density chrominance modulation screen is its high gray scale. Compared to conventional screens with high gray levels and high pixel density, this setup improves information utilization efficiency

The proposed light-field image display system achieves a full-parallax, high-fidelity color display with a horizontal FOV of 98.2° and a vertical FOV of 97.7°, a spatial resolution of 1000 × 1000, a display depth of 45 cm, and a display area of 12.5 inches × 12.5 inches. A full-parallax light field display using two 8 K LCDs achieves 3,751 parallax images^17^. A horizontal parallax light field display with time-sequential directional backlighting provides 192 parallax images^15^. The system proposed in this paper reaches 10,000 parallax images, delivering high-quality display performance and significantly improving information utilization efficiency compared to conventional solutions. The experimental results of the static light-field image display system demonstrated that the chrominance-luminance separated light-field display method can improve information utilization efficiency and enhance light-field display quality. Additionally, it is feasible to construct a dynamic light-field display system using this method. Chrominance and luminance information are controlled separately by two dynamic screens, as shown in Fig. 7. In contrast to traditional dynamic light field displays that utilize a single display panel, our approach employs a dual-screen configuration: a low-grayscale, high-pixel-density screen combined with a high-grayscale, low-pixel-density screen to deliver light field information. By integrating the two screens, high-quality dynamic light-field display effects can be achieved. Compared to existing full-parallax display technologies, this system offers significant advantages in terms of viewing angle, display depth, and image clarity, while its structural design supports scalable manufacturing. As a result, it shows strong potential for applications in areas such as 3D topographic models and cultural artifact exhibitions. Nevertheless, the proposed display systems still face certain limitations, with noticeable noise present in the rendered images. This may be attributed to errors introduced during the binarization of luminance grayscale images using the discrete dither matrix. Future efforts aimed at optimizing and refining the dither matrix could potentially mitigate this issue.

## Materials and methods

### For the LMF

The process begins with laser exposure, where high-precision laser equipment is used to accurately expose the pre-designed image onto a 0.188 mm thick photosensitive film, which is coated with a 3 µm layer of light-sensitive silver halide. The laser light source used in this process is integrated into a mature laser plotting machine, model WDC-8160, with a laser wavelength of 620–680 nm, compatible with the photosensitive material. During the laser exposure process, parameters such as the scanning speed of the laser head, laser exposure energy, mechanical vibration compensation, laser exposure time, material stretching deformation compensation, and conveyor belt speed need to be adjusted to achieve the output of the intensity modulation image. After exposure, subsequent processes such as development and fixing must be completed. Error testing has shown that the errors introduced by the above processes lead to a controllable minimum pixel size of 3.175 µm on the film, allowing for a maximum pixel density of 8000 DPI for the luminance modulation film.

### For the CMF

In order to adapt the CMF to the 8000 DPI LMF, it is necessary to test the pixel density of the CMF through experiments, given that the LMF is set at 8000 DPI. The process begins by configuring an inkjet printer and removing the black ink. Various chrominance synthesis images are tested for an 80 LPI BAMG, which corresponds to pixel densities of 400 PPI, 480 PPI, 560PPI, 640 PPI, 720 PPI, and 800 PPI, respectively. The light-field synthesis images with different pixel densities are used in the same static light-field image display system to examine their display effects. It was found that when the pixel density of the chrominance modulation film is less than 720 PPI, the light-field display effect significantly improves with increasing pixel density. Therefore, the pixel density of the CMF is selected as 720 PPI.

### For the BAMG

The process begins with the precise machining of the roller using a diamond-tipped cutting tool, designed to achieve nanometer-level accuracy. The diamond tool operates on a high-precision lathe, engraving cylindrical lens array microstructures with a periodicity of 317.5 µm onto the roller’s surface. After engraving, the roller undergoes ultrasonic cleaning to remove any residual material, ensuring the quality of subsequent imprinting. Next, a PET substrate film with a thickness of 188 µm is coated with a 200 µm layer of UV-curable resin. The roller, now cleaned, is brought into contact with the UV-coated PET film in a precision imprinting setup. By carefully controlling the imprinting pressure and rolling speed, the microstructures on the roller are accurately transferred onto the UV resin layer of the film. This imprinting process must be conducted in a cleanroom environment to prevent contamination by particulates, which could compromise the integrity of the microstructures. Finally, the UV resin is cured using a UV light source. This step ensures that the cylindrical lens structures within the UV resin are stabilized and permanently fixed onto the PET film.

### For the CLS

A 3 mm × 3 mm LED chip is used to stably emit white light. A Fresnel lens with a diameter of 40 cm is employed to collimate the white light emitted by the LED chip. To ensure that the divergence angle of the collimated light beam remains sufficiently small, the focal length of the Fresnel lens is set to 30 cm, and it is positioned exactly 30 cm above the LED chip, aligning the distance between the LED chip and the Fresnel lens with the focal length. As a result, the white light emitted by the LED chip is collimated by the Fresnel lens, forming a uniform light source with a divergence angle of less than 2°. Light of different colors can all be collimated to below 2°, making the chromatic aberration imperceptible to the human eye.

### For system assembly

The chromatic modulation film and the luminance modulation film must be precisely aligned, otherwise issues such as artifacts, color shift, and reduced contrast may occur. During film preparation, a crosshair is used to calibrate the films. The two films are separately attached to upper and lower vacuum suction platforms. Alignment is achieved by adjusting the platforms while scanning the crosshair calibration points under a microscope. The films are then bonded using UV glue, enabling the coordinated modulation of chromatic and luminance information.

## Supplementary information

visualization 1

visualization 2

visualization 3

## Data Availability

The corresponding author can provide the supporting data upon reasonable request.
